# Evaluation of p53 Expression in Megakaryocytes Enhances Detection of TP53 Mutations and Residual Disease in Myeloid Neoplasms

**DOI:** 10.7759/cureus.110130

**Published:** 2026-06-02

**Authors:** Linlin Gao, Jonah S Rong, Da Zhang, Janet Woodroof, Wei Cui

**Affiliations:** 1 Department of Pathology and Laboratory Medicine, Veterans Affairs (VA) Medical Center, Kansas City, USA; 2 Science, Pembroke Hill School, Kansas City, USA; 3 Department of Pathology and Laboratory Medicine, The University of Kansas Cancer Center, Kansas City, USA; 4 Department of Pathology and Laboratory Medicine, The University of Kansas Medical Center, Kansas City, USA

**Keywords:** acute myeloid leukemia, immunohistochemistry, myelodysplastic syndrome, myeloid neoplasm, p53, residual disease, tp53

## Abstract

Background and objective: Patients with *TP53*-mutated myeloid neoplasms have poor clinical outcomes, and studies evaluating p53 expression by immunohistochemistry (IHC) in treated disease are limited. This is the first study to assess p53 expression in megakaryocytes (MKs) compared with total bone marrow cells (TBMCs) and to examine its association with *TP53* mutation status in both newly diagnosed/recurrent myeloid neoplasms and treated cases with residual disease.

Methods: We evaluated 214 patients, including 130 with acute myeloid leukemia (AML), 61 with myelodysplastic neoplasm (MDS), and 23 negative controls. p53 expression by IHC was assessed in MKs and TBMCs and correlated with *TP53* mutational status.

Results: p53 expression in both MKs and TBMCs was significantly higher in *TP53*-mutated myeloid neoplasms than in *TP53* wild-type cases and negative controls at diagnosis/recurrence. Evaluation of MKs improved IHC specificity from 0.91 to 0.98. In treated *TP53*-mutated cases, p53 expression in MKs and TBMCs correlated well with genetic and flow cytometry findings, with MK assessment also slightly improving sensitivity.

Conclusion: p53 IHC is a rapid, cost-effective, and reliable tool for detecting *TP53* aberrations and residual disease in myeloid neoplasms, particularly in settings with limited access to molecular testing. Concurrent evaluation of p53 expression in MKs and TBMCs improves both diagnostic specificity and sensitivity.

## Introduction

The tumor suppressor p53 plays a critical role in preserving genomic integrity and cellular stability by regulating cell cycle arrest, facilitating DNA repair, and promoting apoptosis. The *TP53* gene is one of the most frequently altered genes in human cancers [1-4]. *TP53* mutations are present in ~5-10% of myelodysplastic neoplasms (MDS) and ~7-15% of de novo acute myeloid leukemia (AML) but are more common in therapy-related disease, reaching frequencies of up to 40% [5-7]. The classifications of MDS with biallelic *TP53* inactivation and myeloid neoplasms with mutated *TP53* were introduced in the 2022 World Health Organization (WHO) and International Consensus Classifications (ICC) [8,9]. In AML and MDS, *TP53* mutations are associated with adverse cytogenetics and unfavorable clinical outcomes [10-13]. Furthermore, *TP53*-mutated AML and MDS with increased blasts share similar molecular characteristics and clinical outcomes. They should be considered a distinct molecular disease entity [14].

p53 protein expression has been utilized as an indirect indicator of *TP53* mutation in MDS and AML [12,15-17]. In both MDS and de novo AML patients, a p53 expression level of 3+ nuclear staining in at least 5% of bone marrow cells (BMCs) was established as the threshold for clinical significance [18-20]. A recent study showed the utilization of p53 immunohistochemistry (IHC) to detect residual disease in *TP53*-mutated AML and MDS patients, in which more than 10 cells with 2-3+ nuclear p53 staining in any one 400× field were defined as positive [21].

Studies on p53 expression by immunostaining to evaluate residual disease in AML and MDS are relatively limited but offer an opportunity for rapid assessment of residual disease [21]. Post-chemotherapy-treated bone marrow specimens are typically hypocellular, presenting a challenge for residual disease evaluation. Megakaryocytes (MKs) are the largest hematopoietic cells in the bone marrow. In this study, we evaluated whether incorporating p53 staining assessment in both total bone marrow cells (TBMCs) and MKs improves the detection of *TP53* aberrations in myeloid neoplasms across both newly diagnosed or recurrent cases and treated specimens with residual disease.

This work was submitted as a meeting abstract for presentation at the 2026 Annual Meeting of the College of American Pathologists (CAP) in October 2026. Final decision is pending.

## Materials and methods

Case selection

We retrospectively identified 214 patients using a keyword search in our electronic medical record system from 2015 to 2024. The cases included patients with newly diagnosed or recurrent AML (n=90), newly diagnosed or recurrent MDS (n=56), and treated *TP53*-mutated AML and MDS at various time points (n=45). AML or MDS cases with available cytogenetics and molecular genetics were included in the study. Additionally, lymphoma cases with negative staging bone marrow results were included as a negative control group (n=23). Cases lacking cytogenetic and molecular genetic information were excluded. Specimens with fewer than 20 assessable MKs were also excluded. All diagnoses were made according to the 2022 WHO classification [8]. Clinical, morphologic, immunophenotypic, molecular, and cytogenetic data were reviewed. This retrospective study was approved by the Institutional Review Board (IRB), and the requirement for patient consent was waived.

IHC staining and digital image analysis

IHC analysis was performed on bone marrow core biopsies or cell clots using formalin-fixed, paraffin-embedded tissue sections. Expression of p53 was assessed using the p53 (DO-7) mouse monoclonal antibody (Agilent Dako, Santa Clara, CA, USA). The primary antibody was used at the following concentration: p53 (ready to use, undiluted). The Aperio ScanScope system was used to scan the slides stained with p53, and the nuclear algorithm of the Aperio ImageScope software (Aperio Technologies, Vista, CA, USA) was used to analyze the staining. The staining intensity was assessed using a scale of 0-3 (0: no staining; 1+: weak; 2+: moderate; 3+: strong). In each case, at least five representative marrow areas were selected from different regions of the bone marrow section, focusing on well-preserved areas with adequate cellularity and avoiding regions with significant crush artifact or staining artifacts. More than 200 nucleated marrow cells were evaluated in each selected area. In addition, at least 20 evaluable MKs were assessed when available. An average of 59 MKs were analyzed per case (median, 53; range, 20-197). Any positive cells determined by the software algorithm were included. Cases with challenging morphology, including treated specimens, were re-evaluated by an experienced pathologist to ensure the quality and consistency of the analysis.

We used a cutoff of ≥5% 3+ nuclear staining of p53 to define positive protein expression in total cells [18-20]. For MKs, positive p53 expression was defined as ≥4.17% 3+ nuclear staining, based on the Youden index, which was established through receiver operating characteristic (ROC) analysis on negative control cases and MDS and AML cases with wild-type (WT) or aberrant *TP53*. In a subset of treated cases with marked hypocellularity, more than 10 2-3+ p53-expressing cells in any one 400× field were alternatively defined as positive protein expression in total cells [21].

Molecular analysis, conventional cytogenetic studies, and FISH

Cytogenetic testing, either by conventional karyotyping or by fluorescence in situ hybridization (FISH) for loss of chromosome 17, 17p deletion, or chimerism, was performed on diagnostic, relapsed, and treated specimens. The conventional karyotypes were reported according to the 2024 International System for Human Cytogenetic Nomenclature [22]. The data were collected from our electronic medical records.

Nucleic acid was purified from fresh bone marrow or peripheral blood samples using the QIAamp DNA Blood Mini Kit (Qiagen, Germantown, MD, USA). Forty nanograms of input DNA underwent a multiplex polymerase chain reaction (PCR) reaction targeting all coding exons within 141 myeloid-related genes using the QIAseq Targeted DNA Human Myeloid Neoplasms Panel or a *TP53*-specific panel that covers exons 2-12 of the *TP53* gene (Qiagen) [23]. Prepared libraries were subjected to next-generation sequencing (NGS) on a NextSeq 500 instrument (Illumina, San Diego, CA, USA) to generate FASTQ files. Reads were mapped to the GRCh37 reference genome using the CLC Genomics Workbench (Qiagen) to generate Variant Call Files, which were processed using Clinical Insight-Interpret (Qiagen) to assess pathogenicity based on American College of Medical Genetics and Genomics and Association for Molecular Pathology (ACMG-AMP) guidelines [24]. Quality control metrics such as depth of coverage, variant allele frequency (VAF), and average quality scores for reported variants were evaluated individually for Pathogenic and Likely Pathogenic calls compared with Variants of Uncertain Significance (VUS). The analytic sensitivity was 95.0% for detecting single-base substitutions and insertion/deletion events with an allele frequency >5% for the Targeted DNA Human Myeloid Neoplasms Panel and 1.0% allele frequency for the *TP53*-targeted panel.

Statistic analysis

Student's t-test was performed using Microsoft Excel software (Microsoft, Richmond, VA, USA). A p-value <0.05 was considered statistically significant. Positive p53 expression in MKs was defined using the Youden index, which was derived from ROC analysis (IBM SPSS, Chicago, IL, USA).

## Results

Evaluation of p53 expression in MK and TBMC populations in newly diagnosed and recurrent myeloid neoplasms

**Figure 1:**
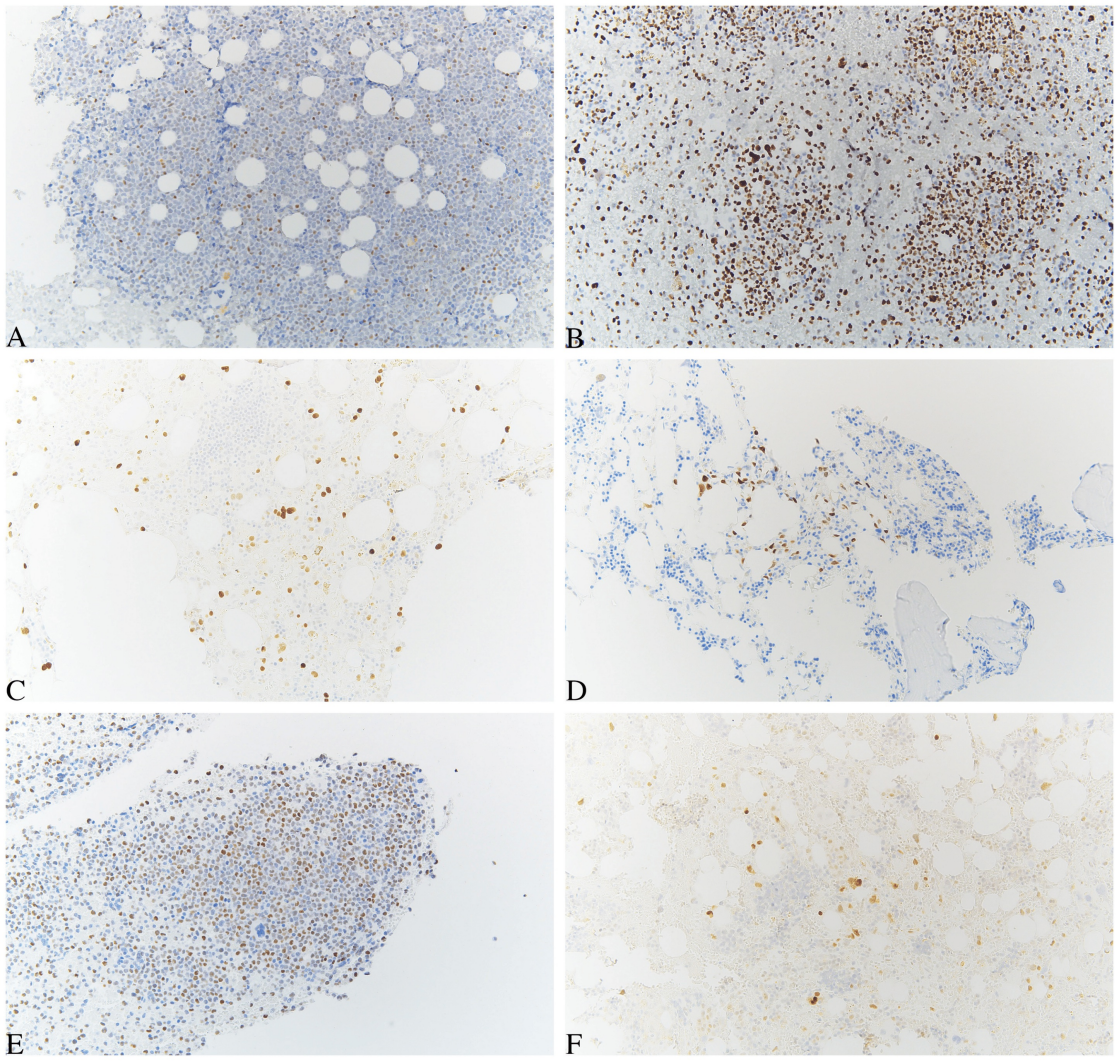
p53 expression in patients with myeloid neoplasms (A) Wild-type *TP53* AML patient at diagnosis (200×). (B) *TP53*-mutated AML patient at diagnosis (200×). (C) Treated a *TP53*-mutated AML patient with p53 overexpression in both MKs and TBMCs (200×). (D) Treated a *TP53*-mutated AML patient with p53 overexpression in a single focus of more than 10 cells with 2-3+ nuclei (200×). (E) Treated a *TP53*-mutated AML patient with p53 overexpression in TBMCs only (200×). (F) Treated a *TP53*-mutated MDS patient with p53 overexpression in MKs only (200×). MKs, megakaryocytes; TBMCs: total bone marrow cells; AML, acute myeloid leukemia; MDS, myelodysplastic neoplasm

p53 expression in both MK and TBMC populations was evaluated in newly diagnosed or recurrent AML (n=90), MDS (n=56), as well as in negative lymphoma staging cases (n=23). The mean and median percentages of 3+ nuclear p53 expression in both MK and TBMC populations were significantly higher in AML and MDS cases with *TP53* mutations compared with WT *TP53* cases and the negative control group (Table 1; p-values <0.0001). WT p53 expression was observed in both MK and bone marrow cell populations in all negative controls, as well as in the majority of AML (n=49, Figure 1A) and MDS (n=43) cases with WT *TP53*. In contrast, diffuse p53 overexpression was identified in both MK and total marrow cell populations in AML (n=28, Figure 1B) and MDS (n=8) cases harboring *TP53* aberrations.

**Table 1:** p53 (3+ intensity) expression in MK and TBMC populations across patient cohorts *Negative control vs. mutant *TP53*; ^†^wild-type *TP53* vs. mutant *TP53*; ^‡^mutant *TP53* vs. discordant; ^#^wild-type *TP53* vs. discordant p-values were calculated using the Student's t-test in Microsoft Excel (Microsoft, Richmond, VA, USA). MK, megakaryocytes; TBMC, total bone marrow cell

	Negative control (n=23)		Wild-type *TP53* (n=92)		Mutant *TP53* (n=36)		Discordant (n=18)	
	MK	TBMC	MK	TBMC	MK	TBMC	MK	TBMC
Mean	0	0.13	0	2.4	29.1	38.4	4.8	7.4
Median	0	0	3.9	0.24	28.3	40.2	0	7.3
p-value	<0.0001*	<0.0001*	<0.0001^†^	<0.0001^†^	<0.0001^‡^	<0.0001^‡^	0.13^#^	0.0003^#^

There were 18 discordant cases, including WT *TP53* AML (n=9) and MDS (n=5) cases showing p53 overexpression in MKs (n=3), TBMCs (n=9), or both (n=2), as well as *TP53*-mutated AML cases with a null p53 immunophenotype (n=3). One case of *TP53*-mutated AML showed p53 overexpression in TBMCs, but not in MKs, at diagnosis (n=1). At the time of persistent disease, p53 overexpression was observed in both MKs and TBMCs.

Sensitivity and specificity for detecting *TP53* aberrations using positive p53 expression in MKs, TBMCs, or both as surrogate markers

Sensitivity and specificity for detecting *TP53* aberrations using positive p53 expression were evaluated (Table 2). Three AML cases with a p53 null phenotype were excluded. When p53 expression was assessed in MKs alone, the sensitivity and specificity were 0.97 and 0.96, respectively. For analysis based on TBMC populations, the sensitivity was 1.0, but the specificity decreased to 0.91. Combining p53 expression analysis in both MKs and TBMCs maintained the same sensitivity (1.0) while improving the specificity to 0.98 (Table 2).

**Table 2:** Sensitivity and specificity of p53 IHC across cell types in myeloid neoplasms at diagnosis/recurrence MK, megakaryocytes; TBMC, total bone marrow cells; TP, true positive; TN, true negative; FP, false positive; FN, false negative; IHC, immunohistochemistry

	p53-3+ (MK+TBMC)	p53-3+ (MK)	p53-3+ (TBMC)
TP	36	36	37
TN	115	124	118
FP	2	5	11
FN	0	1	0
Sensitivity	1	0.97	1
Specificity	0.98	0.96	0.91

Evaluation of p53 expression in MKs and TBMC populations in treated myeloid neoplasms

p53 expression in MKs and TBMC populations was subsequently evaluated in treated AML, including one case of blast-phase chronic myeloid leukemia (n=40), and MDS (n=5) cases with *TP53* aberrations (Table 3), some of which were longitudinal samples obtained from the same patients at different time points during treatment. The criteria used to define positive p53 expression in MKs remained the same. However, positive p53 expression in TBMCs was defined by either >5% of 3+ nuclei or more than 10 cells with 2-3+ nuclei in any one 400× field of the specimen because of the paucicellularity of treated specimens [21].

**Table 3:** Patient information for treated TP53-mutated AML and MDS cases ^*^AML-screen panel; ^#^*TP53*-targeted panel IHC, immunohistochemistry; AML-MR, acute myeloid leukemia, myelodysplasia-related; MDS-LB, myelodysplastic neoplasm with low blasts; MDS-IB, myelodysplastic neoplasm with increased blasts; CML, chronic myeloid leukemia; NP, not performed; NA, not applicable; +/-, atypical; DS, detection sensitivity; IS, insufficient; Con, consistent; Dis, discrepant; AML, acute myeloid leukemia; MDS, myelodysplastic neoplasm

Case	WHO diagnosis	Cytogenetics	NGS	VAF (%)	Flow	IHC vs. other	Cellularity	Blasts (%)	p53- total	p53- MKs
1-1	AML-MR	FISH: 3.8% female recipient; 41~43,XX,add(4)(q12),add(5)(q31),del(5)(q13q33),add(7)(q11.2),del(8)(p21),-12,-14,-17,~17, add(19)(q13.1),-21,add(21)(p11.2)add(22) (q11.2),+1~3mar(4)/46,XY(16)	NP	NA	+	Con	30	<1	+	+
2-1	AML-MR	41-44,XX,add(5)(q11.2),del(7)(q11.2q32),der(8)t(6;8)(p21:q24), der(12;13)(p11.2;p13)ins(12:?) (p11.2;?),-16,der(17:18)(q10;q10),del(18)(q12q21.1),-19,add(19)(p13.3),+0-1mar(20)	NP	NA	+	Con	30	53	+	+
2-2		42~44,XX,-3,del(5)(q13q35), del(7)(q11.2q32),der(8)t(6;8)(p21;q24),der(12)t(12;13)(?p11.2;q12),-16,-17,-18,-19,add(19)(p13.3),+1~3mar(7)/46,XX(13)	NP	NA	+	Con	30-40	4	+	+
3-1	AML-MR	44~47,X,-X,del(5)(q13q35),-8,+11,-12,add(17)(p11.2),-18,-19, +2~4mar(8)/46,XX(12)	p.H179R	2.1^#^	+	Con	5-10	2	+	-
4-1	AML-MR	FISH: 13.8% female recipient; 46,XY(20)	NP	NA	+	Dis	20-30	1	-	-
5-1	AML-MR	46,XX(20)	-	0	-	Con	40	1	-	-
6-1	AML-MR	46,XX(20)	p.I195T	10.3^#^	+/-	Con	30	1	+	+
6-2		46,XX(20)	p.I195T	6.0^#^	+	Con	30-40	2	+	-
7-1	AML-MR	41~44,XX,del(5)(q13),dic(9;19)(19pter->19q13.3::9p13->9qter),-13,der(13;17)(q10;q10),-18, add(19)(p13.1),del(20)(q11.2q13.3),+21,dic(21;21)(q22;q22),+1~2mar(4)/46,XX(16)	pR248W	8.8^#^	+	Con	5-10	1	+	+
7-2		42,XX,del(5)(q13),-13,der(13;17) (q10;q10),18,add(19)(p13.1),+psu dic(19;9)(19qter->19p13.3: :?: : 9p11->9qter),del(20)(q11.2q13.3),idic(21)(q22),+2mar(1)/46,XX(9)	pR248W	6.0^#^	-*	Con	10-20	2	+	+
8-1	AML-MR	41~43,X,-X,-3,del(5)(q31q35),-7,add(20)(q11.2),+mar(4)/46,XX(16)	p.R175H	Not available	+	Dis	10-30	2	-	IS
9-1	AML-MR	42~45,XY,t(1;20)(p34.1;q13.3),-4,-5,add(6)(p21.3),add(17)(p11.2),+mar(8)/46,XY(12)	p.M246V	13	+	Con	30-40	2	+	IS
9-2		44-47,XY,-4,-5,+6,add(6)(p21.3)x2,+8,-11,add(17)(p11.2),+r,+mar(10)/45,XY,sl,t(1;20)(p34;q13.3),-add(6) (p21.3),-8,+11,t(12;19)(q21;p13),-r(2)/49,XY, sl,+15,+21(2)/46,XY(6)	p.M246V	6.14	-	Con	5-10	4	+	IS
10-1	AML-MR	46,XX(20)	-	0	-	Con	40-50	3	-	-
10-2		46,XX(20)	p.C176Y	11	+	Con	5-30	2	+	-
11-1	AML-MR	46,XY(20); FISH 17p-/-17: (-)	p.V216M; p.G187S	1.6; 2.6^#^	+	Dis	40	1	-	-
11-2		46,XY(20)	p.G187S	1.0^#^	+	Dis	10-80	1	-	-
11-3		46,XY(20)	p.V216M; p.G187S	0.7; 1.3	-	Dis	40-50	2	-	-
12-1	AML-MR	45,XX,add(4){q21),der(5)t(5;7)(q13;p13),-7,hsr(11)(q23),hsr(21) (p11.2)(1)	NP	NA	-*	Con	5	2	+	+
13-1	AML-MR	42~47,XX,+X,-4,add(4)(p12),-5,+6,add(7)(q11.2),+11,-16, add(17)(p11.1),-18,-20,+mar(1)/XX,46(11)	-	0	-*	Dis	5-30	2	-	-
13-2		46,XY(20)	-	0	+/-	Con	20	0	-	-
14-1	AML-MR	46,XY(20)	-	0	-	Con	40	2	-	-
14-2		44~46,XX,-3,add(5)(q11.2),-7,t(9;13)(q22;q14),del(15)(q11.2q21),der(17)add(17)(p11.2)add(17) (q2?1),+mar(1)/46,XX(14)	NP	NA	+	Con	20	2	+	+
15-1	AML-MR	45,XY,-5,add(17)(q23),-20,+mar(3)/44,XY,sl,der(13)t(13;15)(q32;q15),-15(2)/46,XY(15)	p.Y236C	0.9	+	Con	30-40	3	+	+
16-1	AML-MR	46,XX,der(4)add(4)(p16)add(4)(q31.3),t(4,12)(q21;q15),del(5)(q13q33),+8,-9,hsr(11)(q23), der(17)t(9;17)(p13;p11.2)(4)/46,XX(16)	p.V272M	2	+	Con	40	1	-	+
17-1	AML-MR	46,XX,der(3)add(3)(p25)add(3)(q11.1),-4,del(5)(q13q33),der(6)t(3;6) (q21;p23),add(10)(q22),add(15) (q11.2),add(16)(q22),-17,-20, add(21)(q22),+3mar(14)/46,XX(6)	NP	NA	+	Con	90	66	+	+
17-2		46,XX,der(3)add(3)(p25)add(3) (q11.1),-4,del(5)(q13q33),der(6),t(3;6)(q21;p23),add(10)(q22), add(15)(q11.2),add(16)(q22),-17,-20,add(21)(q22),+3mar(20)	NP	NA	+	Con	90	80	+	+
18-1	AML-MR	46,XY,del(5)(q13q33)(1)/43~46, sl,der(1)t(1;6)(q12;p21),+der(1) t(1;7)(p22;p11.2),7,+10,add(10) (q24)x2,-11,i(11)(q10),-17,+mar(14)/46,XY(5)	NP	NA	+	Con	40-50	28	+	+
18-2		46,XY,del(5)(q13q33)(3)/60,sl,+Y,+der(1)t(1;7)(p22;p11.2),+2,+add(4)(q31),+5,+6,+der(6)t(1;6)(p13;q11),-7,+8,+add(10)(q24)x3,-11,i(11)(q10),+13,+15,+20,+mar(1)/46,XY,del(5)(q21q34)(7)/45,XY,-7(1)/46,XY(8)	p.K139_P142del	63	+	Con	50	5	+	+
19-1	AML-MR	46,XY(20); FISH -17 and 5q-: (-)	p.Y220C	1.8	+	Dis	40-50	4	-	-
20-1	AML-MR	33~48,XY,-4,del(4)(q31.1),-5,add(7)(q11.2),-9,-11,-+20,-21,add(21)(p11.2),i(21)(q10),-22,+r,+1~5mar(5)/46,XY(15)	p.R158L	7	+	Con	20-30	0	+	+
21-1	AML-MR	43-46,XY,add(4)(q25),del(5)(q13q35),-7,del(12)(p11.2),add(12)(q24.1),t(13;17)(q12; p11.2),-18,-20,-21,der(21)t(18;21)(q11.2;p11.2),+2-5mar(15)/86-89,XY,slx2(5)	p.P278R	59	+	Con	100	22	+	+
22-1	MDS-LB	47,XY,+8(1)/43~46,XY,del(5) (q1 3q35),-7,t(13;17)(q12;p11.2),-18,-20,-21,der(21)t(1 8;21) (q11.2;p1 1.2),+1~2r,+1~2mar (14)/41~45,XY,s|,add(3)(q21)(2)/46,XY(3)	NP	NA	+	Con	95	2	+	+
22-2		43~47,XY,del{5)(q13q35),-7, t{13;17)(q12;p11.2),-18,-20,-21, der(21)(18;21)(q11.2;p11.2),+1~2r,+1~4mar(16)/46,XY(4)	NP	NA	+	Con	60-70	4	+	+
22-3		46,XY(20)	NP	NA	-	Con	30-40	1	-	-
23-1	AML-MR	42-44, XY,add(1)(p22),add(2)(p13), add(2)(q37),-3,add(5)(q22),-7, +der(12)t(1;12)(p22;q13),-17,-20, mar(5)/46, XY(15)	-	0	+	Con	40-50	12	+	+
24-1	AML-MR	42~46,XX,add(6)(p23),-7,der(7) inv(7)(p1 5.3q21.2)del(7)(q21.2), add(8)(q21.3),del(9)(q13q31),-11, ins(13;?)(q12;?),add(17)(p12), add(21)(q22.3),del(21)(q22),-22,+1~2mar,+r(20)	NP	NA	+	Con	90-100	81	+	+
25-1	MDS-IB-1	46,XX(20)	-	0	+	Dis	30	<1	-	-
25-2		46,XX,?add(1)(p34),add(5)(q31), del(7)(p13),add(8)(q11.2)(1)/46, XX(19)	-	0	+	Con	20	1	+	+
25-3		46,XX(20)	p.C135Y	63	+	Con	20	1	+	-
26-1	AML-MR	45,XX,dic(10;17)(p11.2;p11.2)(13)/46,XX(7)	NP	NA	-*	Con	90	60	+	-
27-1	Blast phase CML	48~48,XY,+8,t(9;22)(q34;q11.2), i(17)(q10),der(18)t(1;18)(q21;p11.1),+der(22)t(9;22)(4)/46,XY(10)	NP	NA	-*	Con	80	50	+	IS
28-1	AML-MR	41~47,XX,-9,der(9;21) (q10,q10),add(13)(p11.2),-19, add(21)(p11.2),+add(21)(p11.2), +add(21)(p11.2),+2~6mar(20)	NP	NA	+	Con	70	22	+	+
29-1	AML-MR	44~45,XY,-3,-5,der(7)del(7)(q22) inv(7)(p13q22),del(12)(p11.2), add(19)(q13.1),+mar(20)	NP	NA	+	Con	80	20	+	+
30-1	AML-MR	45,XY,-2,-3,-5,-7,-9,del(10) (q24),del(12)(p11.2),-13,-14, add(16)(p11.2),add(17)(p11.2),+r,+4mar(6)/46,XY(1)	p.V274G	26	+	Con	5-80	30	+	+

p53 was overexpressed in both MKs and TBMCs in 23 cases (Figure 1C), while WT p53 expression was present in both MKs and TBMCs in 12 cases. p53 overexpression in TBMCs was detected only in a single focus of more than 10 cells with 2-3+ nuclei in three cases (Figure 1D). Six discordant cases included five cases with overexpressed p53 in TBMCs and WT p53 expression in MKs (Figure 1E) and one case with overexpressed p53 in MKs and WT p53 expression in TBMCs (Figure 1F). There were four cases with overexpressed p53 in TBMCs, but insufficient numbers of MKs for evaluation.

Correlation of p53 expression with cytogenetics, molecular genetics, and flow cytometric studies in treated myeloid neoplasms

Overexpression of p53 in either MKs or TBMCs was concordant with positive findings by cytogenetics, NGS, and flow cytometry in 32 cases, including those with minimal involvement, such as a single abnormal metaphase or a *TP53* mutation detected by NGS with a VAF as low as 0.9%, below the 5% detection sensitivity of the Targeted DNA Human Myeloid Neoplasms Panel (Table 3). WT p53 expression was concordant with negative cytogenetic, NGS, and flow cytometric results in five cases. There were eight discordant cases in which p53 IHC did not correlate with genetic or flow cytometric findings. Notably, four of these cases harbored *TP53* mutation VAFs <3% (range, 0.7%-2.6%), which were either below the 5% detection sensitivity of the Targeted DNA Human Myeloid Neoplasms Panel or near the lower limit of detection (1%) of the *TP53*-targeted panel. In addition, one case showed only a single abnormal metaphase on cytogenetic analysis. Another case demonstrated a normal karyotype and negative NGS findings but a positive flow cytometry result. The *TP53* variants included predominantly missense mutations, with one case harboring an in-frame deletion.

Sensitivity and specificity for detecting residual disease using positive p53 expression in MKs, TBMCs, or both as surrogate markers in treated myeloid neoplasms

Sensitivity and specificity for detecting *TP53* aberrations using positive p53 expression in treated myeloid neoplasms were evaluated (Table 4). The sensitivities were 0.82, 0.79, 0.77, and 0.77 when using positive p53 expression in either MKs or TBMCs, TBMCs alone, MKs alone, or both MKs and TBMCs, respectively (Table 4). The specificity was 1.0 across all combinations.

**Table 4:** Sensitivity and specificity of p53 IHC across cell types in treated myeloid neoplasms MK, megakaryocytes; TBMCs, total bone marrow cells; TP, true positive; TN, true negative; FP, false positive; FN, false negative; IHC, immunohistochemistry

	Flow	p53-3+ (MK+TBMC)	p53-3+ (TBMC)	p53-3+ (MK)	p53-3+ (MK or TBMC)
TP	32	23	31	24	32
TN	4	5	6	5	6
FP	0	0	0	0	0
FN	2	7	8	7	7
Sensitivity	0.94	0.77	0.79	0.77	0.82
Specificity	1	1	1	1	1

Evaluation of flow cytometry findings in treated myeloid neoplasms

AML measurable/minimal residual disease (MRD) flow cytometry assays were performed on 40 cases (Table 3), with total acquired cell events ranging from 84,042 to 500,000. Two cases interpreted as having “atypical immunophenotypic findings” were excluded from the sensitivity and specificity analysis. In five “dry tap” cases, bone marrow core biopsy specimens were submitted for flow cytometric analysis in place of aspirated marrow specimens used in routine cases; therefore, a limited AML screening panel was performed, with total acquired cell events ranging from 1,955 to 12,351. Among the AML MRD studies, 31 cases demonstrated concordant positive results, and four cases demonstrated concordant negative results. Two cases showed false-negative results; in one of these, the false-negative result was attributed to limited cellularity, with only 84,042 total acquired cell events. Two cases yielded equivocal results, and one case demonstrated a positive MRD flow cytometry result despite negative findings by cytogenetics, NGS, and p53 IHC.

All five “dry tap” cases evaluated using the limited flow panel yielded false-negative results (Table 3). The sensitivity and specificity of the AML MRD flow assay were 0.94 and 1.0, respectively (Table 4). Interestingly, five of the seven cases with false-negative flow results demonstrated overexpression of p53 in TBMCs or in both MKs and TBMCs, further underscoring the diagnostic utility of p53 IHC for residual disease detection in AML.

## Discussion

In this study, we evaluated p53 overexpression in MKs and TBMCs as surrogate markers for *TP53* aberrations in cases of AML and MDS at the time of diagnosis or recurrence, as well as for the detection of residual disease in treated myeloid neoplasms. Consistent with previous studies [18-20], we found that p53 overexpression detected by IHC correlated well with the presence of *TP53* aberrations. Sensitivity remained high (0.97-1.00) when p53 expression was assessed in MKs alone, TBMCs alone, or in combination. However, specificity increased from 0.91 to 0.98 when both MKs and TBMCs were analyzed, compared with TBMCs alone.

Similar to a previous finding [21], p53 IHC provided a rapid and cost-effective approach for the evaluation of residual disease in treated myeloid neoplasms. The overall detection sensitivity was 0.82 when p53 IHC was assessed in either MKs or TBMCs. When analyzed separately, the sensitivity was 0.79 for TBMCs and 0.77 for MKs. Importantly, the specificity remained 1.0 across all combinations, indicating a high level of reliability in ruling out *TP53* aberrations when p53 overexpression was absent.

Interestingly, p53 IHC was able to detect low-volume residual disease, including three cases with a single focus of >10 cells showing 2-3+ nuclear staining, one case with an abnormal karyotype in a single cell, and one case with a *TP53* mutation identified by NGS with a VAF of 0.9%. Among the available modalities, the AML MRD flow cytometry assay demonstrated the highest sensitivity (0.94) for detecting residual disease. However, when a limited panel was applied to bone marrow core specimens or when a hypocellular specimen was analyzed, the assay yielded false-negative results. Notably, p53 IHC successfully identified residual disease in five out of these six cases, underscoring its valuable role as a complementary tool to flow cytometry in the detection of residual disease, particularly in challenging specimens such as hypocellular marrows.

Evaluation of p53 expression in both MKs and TBMCs improved the specificity from 0.91 to 0.98 while also resulting in a slight increase in sensitivity for residual disease detection compared with assessing MKs or TBMCs alone.

However, a key limitation of this study was the occasional absence of an adequate number of MKs for evaluation, which led to the exclusion of some cases at the time of diagnosis or recurrence. In particular, four treated cases did not contain a sufficient number of MKs to allow a reliable assessment of residual disease. This highlights an important practical constraint of using MK-based p53 IHC, especially in post-therapy specimens where marrow cellularity and lineage representation may be markedly reduced. Another limitation is the requirement to perform p53 IHC on all diagnostic specimens to exclude a “null” expression pattern, which may otherwise confound interpretation of subsequent results. In addition, the retrospective nature of this study may introduce inherent limitations related to case selection, variable specimen quality, and incomplete clinical or laboratory data. As a single-institution study, institutional referral patterns and testing practices may also introduce selection bias and limit the generalizability of these findings to other practice settings.

## Conclusions

In summary, p53 IHC is a rapid, cost-effective, and reliable method for detecting *TP53* aberrations and residual disease in myeloid neoplasms, particularly in settings with limited access to genetic or molecular testing. When p53 expression is evaluated in both MKs and TBMCs, specificity and sensitivity are both improved. Additionally, p53 IHC serves as a valuable adjunct for residual disease assessment, especially in cases where limited or suboptimal specimens may lead to false-negative results by flow cytometry or molecular studies.
